# The Effect of the Fiber Diameter, Epoxy-to-Amine Ratio, and Degree of PVA Saponification on CO_2_ Adsorption Properties of Amine-Epoxy/PVA Nanofibers

**DOI:** 10.3390/polym17141973

**Published:** 2025-07-18

**Authors:** Chisato Okada, Zongzi Hou, Hiroaki Imoto, Kensuke Naka, Takeshi Kikutani, Midori Takasaki

**Affiliations:** 1 Nitto Denko Corporation, Ibaraki 567-8680, Japan; 2 Faculty of Molecular Chemistry and Engineering, Kyoto Institute of Technology, Kyoto 606-8585, Japan; himoto@kit.ac.jp (H.I.); kenaka@kit.ac.jp (K.N.); 3 Faculty of Materials Science and Engineering, Kyoto Institute of Technology, Kyoto 606-8585, Japan; houzongzi@gt.cn; 4 School of Materials and Chemical Technology, Institute of Science Tokyo, Tokyo 152-8550, Japan; kikutani.t.aa@m.titech.ac.jp; 5 Center for Fiber and Textile Science, Kyoto Institute of Technology, Kyoto 606-8585, Japan; 6 Faculty of Environment and Information Sciences, Yokohama National University, Yokohama 240-8501, Japan; takasaki-midori-jy@ynu.ac.jp

**Keywords:** carbon capture, nanofiber, amine-epoxy nanofibers, electrospinning, poly(vinyl alcohol)

## Abstract

Achieving carbon neutrality requires not only reducing CO_2_ emissions but also capturing atmospheric CO_2_. Direct air capture (DAC) using amine-based adsorbents has emerged as a promising approach. In this study, we developed amine-epoxy/poly(vinyl alcohol) (AE/PVA) nanofibers via electrospinning and in situ thermal polymerization. PVA was incorporated to enhance spinnability, and B-staging of AE enabled fiber formation without inline heating. We systematically investigated the effects of electrospinning parameters, epoxy-to-amine ratios (E/A), and the degree of PVA saponification on CO_2_ adsorption performance. Thinner fibers, obtained by adjusting spinning conditions, exhibited faster adsorption kinetics due to increased surface area. Varying the E/A revealed a trade-off between adsorption capacity and low-temperature desorption efficiency, with secondary amines offering a balanced performance. Additionally, highly saponified PVA improved thermal durability by minimizing side reactions with amines. These findings highlight the importance of optimizing fiber morphology, chemical composition, and polymer properties to enhance the performance and stability of AE/PVA nanofibers for DAC applications.

## 1. Introduction

Carbon dioxide (CO_2_) is said to be the gas that has the greatest impact on global warming [1,2,3]. Since the Industrial Revolution [4], CO_2_ emissions have continued to increase due to the large-scale use of fossil fuels such as oil, coal, and natural gas. In particular, high-concentration CO_2_ is contained in large quantities in exhaust gas from thermal power plants and factories. Amine absorption has long been used as a method to capture CO_2_ from the exhaust gas of high CO_2_ concentration and reduce carbon emissions [5,6]. However, simply capturing CO_2_ from exhaust gas is not enough to achieve carbon neutrality by 2050 [7]. It is essential to capture CO_2_ that has been released into the atmosphere in the past and make it carbon negative. Therefore, direct air capture (DAC) [8] has attracted attention as a technology that can directly capture CO_2_ already emitted into the atmosphere.

DAC is a technology that adsorbs very dilute CO_2_ in the atmosphere (currently around 400 ppm) onto an adsorbent and then captures it by heating and reducing pressure. After capturing CO_2_, methods such as highly concentrating it and storing it underground using CCS (Carbon Capture and Storage) technology can be applied. Presently, the most promising approach for DAC is to apply liquid amines to porous materials such as mesoporous silica [9]. The high specific surface area of porous materials allows the chemical adsorption of amines to work more effectively [10]. In addition, applying liquid amines to porous materials allows them to be treated like solid materials. However, amines have problems such as being easily volatilized at high temperatures, being easily washed away by moisture, having low heat resistance, and being easily oxidized [11,12]. This is because liquid amines generally contain a large number of primary amines, which have low molecular weights and are easily degraded by oxidation reactions. In addition, primary amines are highly reactive and easily adsorb CO_2_, but they require high energy for desorption due to their high desorption temperature [13]. To address these challenges, various approaches have been investigated to enhance CO_2_ capture performance [14,15,16]. For example, amine-functionalized adsorbents with strong chemisorption capabilities [17], amine-modified metal–organic frameworks (MOFs) with high surface areas [18], silica materials grafted with amines [19], and oxide supports impregnated with amines [20] have all been explored.

In our previous reports, the fabrication of amine-epoxy/poly(vinyl alcohol) (AE/PVA) fibers with an average diameter of approximately 400 nm was achieved via in situ thermal curing during the electrospinning process [21]. Alternatively, the AE/PVA nanofibers were successfully prepared without the need for inline heating by controlling the B-stage of the amine-epoxy reaction. The resulting fibers exhibited diameters ranging from 500 to 700 nm. Using a varnish solution with a low B-stage degree, it was difficult to maintain the fibrous morphology through electrospinning alone, and inline thermal curing was required [22].

In this report, we investigated the effect of fiber diameter, epoxy-to-amine ratio (E/A), and degree of PVA saponification on CO_2_ adsorption properties of AE/PVA nanofiber webs prepared through electrospinning to explore the optimum conditions for fabricating the absorbent for DAC. Firstly, we investigated how variations in electrospinning conditions and formulation of AE/PVA nanofibers influence their CO_2_ adsorption performance. For neat PVA materials, it has been reported that fiber diameter can be controlled by adjusting the concentration of the aqueous PVA solution, applied voltage, and the distance between the nozzle and the collector [23,24]. Based on this, the authors employed the same spinning solution for the AE/PVA system and systematically varied the applied voltage and the syringe-to-collector distance to examine their effects on the fiber diameter and CO_2_ adsorption behavior.

Furthermore, the authors explored the effect of varying the E/A from 0.3 to 0.55, which had previously been fixed at 0.5, to balance thermal stability and adsorption/desorption kinetics. Prior studies have shown that the E/A influences a trade-off among adsorption capacity, adsorption rate, and low-temperature desorption, depending on the degree of amine substitution.

Lastly, we also examined the influence of the degree of saponification of PVA, which was introduced to improve spinnability. The terminal group composition of PVA varies depending on its degree of saponification, with lower saponification resulting in a higher proportion of acetate groups. It is known that under thermal conditions, such as in situ thermal curing in the spinning process or heat treatment of prepared webs, these acetate groups react with secondary amines to form amide bonds [25,26,27].

## 2. Materials and Methods

### 2.1. Materials

**Figure 1:**
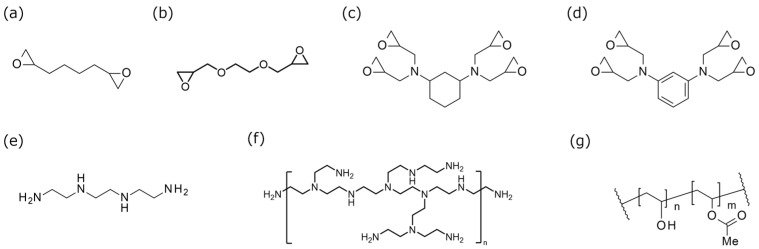
Chemical structural formula of (**a**) 1,7-octadiene diepoxide (ODE), (**b**) ethylene glycol diglycidyl ether (EDE), (**c**) 1,3-bis (*N*,*N*-diglycidyl aminomethyl) cyclohexane (T-C), (**d**) *N*,*N*,*N*′,*N*′,-tetraglycidyl-*m*-xylenediamine (T-X), (**e**) triethylenetetramine (TETA), (**f**) polyethyleneimine (PEI), and (**g**) poly(vinyl alcohol) (PVA).

The chemical structures of the materials used to manufacture the nanofibers are shown in Figure 1a–g. As an epoxy, 1,7-octadiene diepoxide (ODE) (purity > 97%, Tokyo Chemical Industry Co., Ltd., Tokyo, Japan), ethylene glycol diglycidyl ether (EDE) (purity > 99%, EX-810, Nagase ChemteX Corporation, Osaka, Japan), 1,3-Bis (*N*,*N*-diglycidyl aminomethyl) cyclohexane (T-C) (purity > 98%, TETEAD-C, Mitsubishi Gas Chemical Co., INC., Tokyo, Japan), and *N*,*N*,*N*′,*N*′-tetraglycidyl-*m*-xylenediamine (T-X) (purity > 98%, TETRAD-X, Mitsubishi Gas Chemical Co, INC., Tokyo, Japan) were used. As an amine, triethylenetetramine (TETA) (purity > 99%, Tokyo Chemical Industry Co., Ltd., Tokyo, Japan) and polyethyleneimine (PEI) (purity 98–100%, SP-006, Nippon Shokubai Co., Ltd., Tokyo, Japan) were used.

It is well known that upon heating, a mixture of amine and epoxy undergoes a reaction where primary amines are converted to secondary amines, accompanied by crosslinking-induced solidification, thereby enhancing the thermal stability of the amines. Additionally, to impart spinnability to the spinning solution, the prepared amine-epoxy mixture was blended with an aqueous solution of poly(vinyl alcohol) (PVA) (PVA-117: purity > 93%, molecular weight 76,000, degree of saponification 99%; PVA-217: purity > 94%, molecular weight 83,000, degree of saponification 88%, Kuraray Co., Ltd., Tokyo, Japan) shown in Table 1. The molecular weight of PVA was calculated based on the degree of polymerization and the degree of saponification provided in the catalog. The degree of polymerization was expressed as *m* + *n*, where *m* represents the number of saponified units and *n* the number of residual acetate units. The degree of saponification was defined as:

*Degree of saponification* (%) = *m*/(*m* + *n*) × 100 (1)

Using these parameters, the molecular weight *M* of PVA was estimated by the following equation:

*M* = *m*·*M*_*vinyl alcohol*_ + *n*·*M*_*vinyl acetate*_ (2)

where *M*_vinyl alcohol_ = 44.05 g/mol and *M*_vinyl acetate_ = 86.09 g/mol are the molecular weights of the vinyl alcohol and vinyl acetate units.

The amine value is an index indicating the amount of amine groups (-NH_2_, -NHR, -NR_2_) present in a substance, typically expressed in milligrams of KOH equivalent per gram of sample. Regarding the amines used in this study, the amine equivalent of TETA is 27.3 g/mol. According to the catalog value, the polyethyleneimine has a molecular weight of 600, an amine content of 20 mmol/g, and a molar ratio of 35:35:30 for the primary, secondary, and tertiary amines obtained by NMR analysis. The primary amine equivalents in polyethyleneimine were estimated to be 142.9 g/mol based on the amine content of 20 mmol/g and the composition of the primary amine of 35%. Meanwhile, the epoxy equivalent weight was calculated by dividing the molecular weight of the epoxy by the number of epoxy functional groups. Based on this value, the primary amine value is determined, and the amount of epoxy required to react with half (0.5 equivalent) of the primary amines is subsequently calculated. The epoxy equivalents of ODE, EDE, T-C, and T-X were 71.1 g/mol, 87.1 g/mol, 102.0 g/mol, and 99.0 g/mol, respectively.

### 2.2. Electrospinning Equipment and Conditions

**Figure 2:**
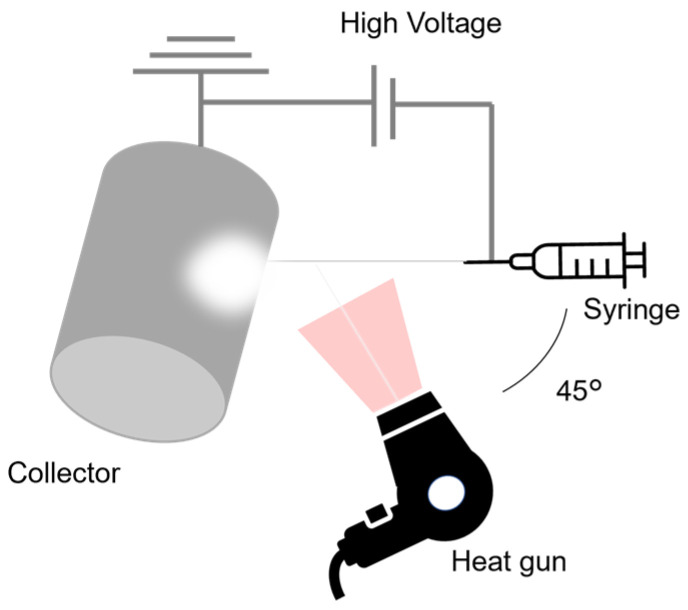
A schematic illustration of the electrospinning apparatus. A heat gun is positioned between the syringe and the collector, angled at 45°, to direct heat toward the fiber formation zone. Fibers are collected on a cylindrical collector.

Electrospinning was conducted using an electric field spinning apparatus (NEU, Kato Tech, Kyoto, Japan) equipped with a fixed cylindrical collector, as shown in Figure 2. Initially, the prepared amine-epoxy/PVA solution was loaded into a 5 mL syringe fitted with a 22 G needle, and electrospinning was performed at an extrusion rate of 0.46–1.36 mL/h, a nozzle–collector distance of 80–200 mm, and an applied voltage of 8–20 kV. A heat gun was employed to thermally enhance the in situ polymerization of the amine-epoxy system during the spinning process, following the approach described in our previous study [14]. The air flow from the heat gun was directed at a 45-degree angle relative to the axis between the spinning solution ejection needle and the collector, at a speed of 3.48 m/min.

### 2.3. Analysis of the Prepared Web

#### 2.3.1. Scanning Electron Microscopy (SEM)

The morphology of the prepared web was examined using a scanning electron microscope (SU-3800, HITACHI High-Tech Co., Tokyo, Japan) at magnifications of ×1000, ×3000, ×5000, and ×10,000. The mean fiber diameter was quantified from the acquired images using image analysis software (ImageJ (version 14.6r)).

#### 2.3.2. Infrared Absorption Analysis

The evolution of the chemical reaction was monitored through the analysis of functional group alteration using an FT-IR spectrometer (Nicolet iS5, FT-IR, Thermo Fisher Scientific Inc., Waltham, MA, USA).

#### 2.3.3. BET Specific Surface Area

The BET specific surface area is the surface area of a fiber web per unit mass analyzed based on the amount of gas adsorption (the BET method). The measurement was conducted using a surface area and porosity analyzer (TriStar II Plus 3030, Micromeritics Inc., Norcross, GA, USA), with krypton as the analysis gas.

#### 2.3.4. Adsorption/Desorption Test

**Figure 3:**
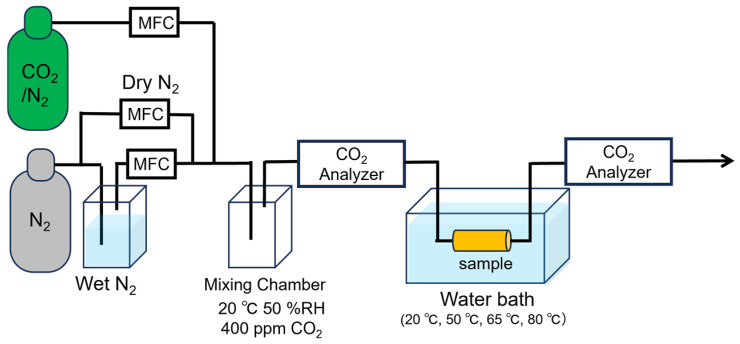
A schematic diagram of the CO_2_ adsorption test apparatus. Gas cylinders containing 10% CO_2_/N_2_ and pure N_2_ are connected to mass flow controllers (MFCs). The gases are bubbled and mixed in a mixing chamber to produce a humidified gas stream at 20 °C and 50% relative humidity (RH), with a CO_2_ concentration of 400 ppm. This gas is delivered to a sample placed in a water bath maintained at 20 °C, 50 °C, 65 °C, or 80 °C. CO_2_ concentrations are monitored before and after the sample using CO_2_ analyzers.

The adsorption and desorption measurement system is schematically represented in Figure 3. The system comprises a 10% CO_2_/N_2_ gas cylinder, a N_2_ gas cylinder, a N_2_ gas humidifier, a gas mixing chamber, a mass flow controller, a gas analyzer for measuring CO_2_ concentration, a sample tube, and a water bath. The dry N_2_ gas supplied from the cylinder is partially humidified via bubbling and subsequently mixed with 10% CO_2_/N_2_ dry gas. The flow rate of each gas is precisely controlled by a mass flow controller and mixed within the gas chamber to achieve a final CO_2_ concentration of 400 ppm, a temperature of 20 °C, a relative humidity of 50%, and a total flow rate of 300 mL/min. Prior to adsorption/desorption testing, the fabricated fiber web is annealed in a vacuum oven (VOS-310C, EYELA, Tokyo, Japan) at 60 °C for 2 h to remove water and CO_2_. Vacuum drying is performed in a dry room (dew point −60 °C). The vacuum drying process at 60 °C for 2 h is conducted to ensure accurate measurement of the sample weight. Furthermore, during the subsequent CO_2_ adsorption/desorption measurements, which are carried out at room temperature, the sample tends to adsorb CO_2_ upon installation. To prevent this, the sample is subjected to heat treatment at 80 °C for 2 h under a 400 ppm CO_2_/N_2_ atmosphere. These procedures are clearly stated in the manuscript. As for the desorption method, as described, the gas flow rate and gas composition are the same during adsorption and desorption: that is, before and during adsorption and desorption, 400 ppm of CO_2_ is constantly flowing at 20 °C and 50%RH. N_2_ gas purging is not used. A CO_2_/N_2_ mixture containing 400 ppm CO_2_, corresponding to the contemporary atmospheric CO_2_ concentration on Earth, was introduced into a sample tube containing 50 mg of the specimen. Initially, the sample tube was immersed in 80 °C water to facilitate CO_2_ desorption, followed by immersion in 20 °C water for 1000 min to enable CO_2_ adsorption. The CO_2_ concentrations in the inlet and outlet gases were continuously monitored using a CO_2_ analyzer (LI-850, MEIWAFOSIS, Tokyo, Japan). The adsorption amount was determined by integrating the differential CO_2_ concentrations between the inlet and outlet gases throughout the experimental duration. Desorption characteristics were analyzed in a similar manner by tracking the CO_2_ concentration variations after immersing the sample tube in 65 °C water. The desorption ratio was subsequently evaluated based on the quantity of desorbed CO_2_ at 50 °C, 65 °C, and 80 °C over a period of 90 min for each.

The Avrami equation was employed to analyze the adsorption kinetics of the DAC sorbents because CO_2_ adsorption involves complex mechanisms beyond a simple first-order reaction, including surface reactions and internal diffusion. The Avrami model [28] allows the adsorption progress to be expressed as a function of time, making it suitable for evaluating both the adsorption rate and the underlying mechanisms. Furthermore, it provides a high degree of fitting accuracy to experimental data, making it a reliable tool for assessing sorbent performance.

## 3. Results

### 3.1. The Effect of the Fiber Diameter in the CO_2_ Adsorption Test

Samples with varying fiber diameters were prepared by adjusting the applied voltage and the distance between the collector and syringe, using a spinning solution described below. For the prepared nanofiber webs, a CO_2_ adsorption test was performed.

The spinning solution comprised EDE: T-X = 8:2 for AE and AE:PVA-117 at 7 wt% aqueous solution with a 1:10 ratio, resulting in an AE weight ratio in the resultant AE/PVA fiber of 58 wt%. The AE/PVA nanofibers were produced by mixing the spinning solution at room temperature and electrospinning with a heat gun set to 120 °C under inline heating conditions. As shown in Table 2, when the distance was increased and the voltage was high, the resulting fibers were thinner, likely due to the enhanced stretching effect. Conversely, when the distance was short and the voltage was high, the drawing force was strong, and the time to reach the collector was short, resulting in thicker fibers due to insufficient stretching.

**Figure 4:**
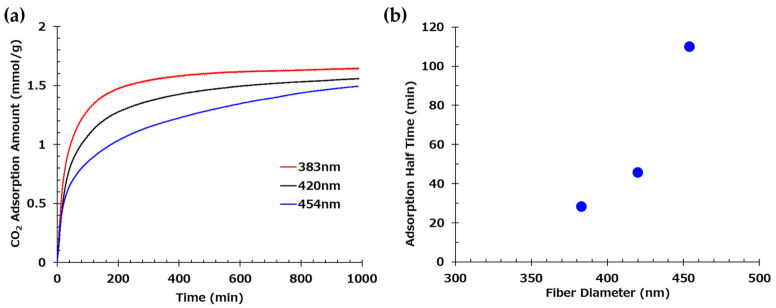
(**a**) Time-dependent CO_2_ adsorption profiles of three web samples with distinct fiber diameters. Measurements were conducted for samples with mean fiber diameters of 383 nm, 420 nm, and 454 nm. (**b**) Correlation between fiber diameter and CO_2_ adsorption half-time.

Figure 4a shows the relationship between time and adsorption amount with respect to fiber diameter, while Figure 4b presents the relationship between the mean fiber diameter and the half-time of adsorption, which is the time it takes to reach half of the saturated adsorption amount, analyzed based on the Avrami model. Thinner fibers exhibited higher adsorption rates. This is attributed to the larger surface area of thin fibers, which allows for greater surface adsorption and reduced effect of internal diffusion of CO_2_ over time. In contrast, thicker fibers are likely to have prolonged adsorption times due to the dominance of internal CO_2_ diffusion, which is time-consuming. These findings indicate that producing AE/PVA nanofibers with a thin and homogeneous fiber diameter is crucial for enhancing adsorption rates.

**Figure 5:**
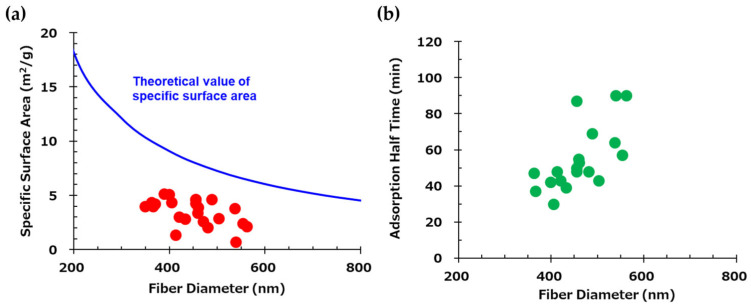
(**a**) Theoretical and experimental values of specific surface area as a function of fiber diameter. The blue curve represents the theoretical values calculated assuming a material density of 1.1 g/cm^3^. Red points indicate experimentally measured values. (**b**) Correlation between the mean fiber diameter and CO_2_ adsorption half-time.

It is generally considered that there is a correlation between fiber diameter and specific surface area. Figure 5a shows the theoretical and experimental specific surface area values as a function of fiber diameter for electrospun fibers. The theoretical curve (blue) was calculated assuming a material density of 1.1 g/cm^3^. Experimental data points (red) were obtained for fibers of various diameters prepared by varying electrospinning parameters, such as applied voltage, syringe tip-to-collector distance, syringe gauge, and solution feed rate, while maintaining the same polymer composition. These results deviate notably from the theoretical values. For instance, in the case of AE/PVA nanofibers with a diameter of 400 nm and a density of 1.1 g/cm^3^, the theoretical BET specific surface area is 9.1 m^2^/g. In contrast, the measured values in this study ranged from 1 to 5.4 m^2^/g. This discrepancy is likely due to the fiber diameter distribution and adhesion between fibers, which reduces the accessible surface area. Adhesion between fibers can be confirmed in the high-magnification SEM images in Table 2.

Figure 5b shows the relationship between the adsorption half-time and the mean fiber diameter. A mild correlation was found between the CO_2_ adsorption rate and fiber diameter. As discussed above, this indicates that the thinner the fiber, the more the amine exposed on the surface contributes directly to CO_2_ adsorption. On the other hand, it is thought that the AE/PVA nanofiber with a thick fiber diameter took longer to adsorb CO_2_ because CO_2_ adsorption by internal diffusion, not just the surface, is dominant. While the adsorption rate increases with thinner fiber diameters, other factors, such as the porosity (density) of the fiber web, may also play a significant role. Higher porosity is believed to facilitate CO_2_ permeance with lower pressure loss and improve CO_2_ adsorption efficiency due to reduced tortuosity.

### 3.2. The Effect of the Epoxy-to-Amine Ratio in the CO_2_ Adsorption/Desorption Test

In the previous sections, to maintain low temperature desorption for superior heat resistance, the ratio of epoxy to amine was fixed with E/A = 0.5 after forming the secondary amines through the chemical reaction of all primary amines with epoxy. In this section, the influence of E/A on spinnability, adsorption/desorption characteristics, and durability was verified by controlling the E/A from 0.3 to 0.55. The spinning conditions were all the same: applied voltage was 10 kV/8 cm, and the temperature of inline heating was 110 °C, for all samples shown in Table 3.

Properties of the obtained fibers are summarized in Table 4. It can be seen that the higher the E/A, the higher the Tg. This is thought to be due to the three-dimensional crosslinking of the amine and epoxy, which strengthens the molecular framework and suppresses molecular motion. In addition, the higher the E/A, i.e., the lower the amount of amine, which is the CO_2_ adsorption site, the saturation adsorption amount in the CO_2_ adsorption test decreases because the epoxy group reacts with the amine.

**Figure 6:**
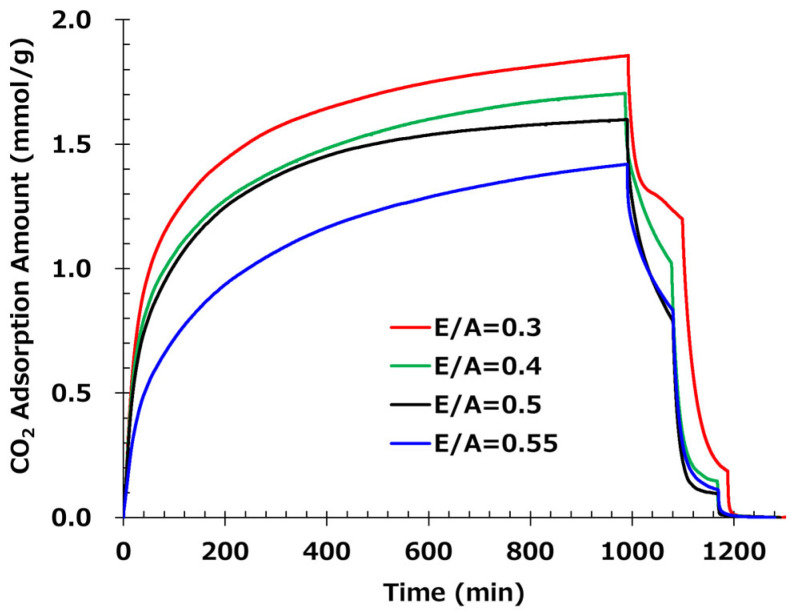
CO_2_ adsorption and desorption profiles of fiber samples prepared with different epoxy-to-amine ratios (E/A). The adsorption amount is plotted as a function of time for E/A of 0.3 (red), 0.4 (green), 0.5 (black), and 0.55 (blue). The temperature is varied at 20 °C for 990 min for adsorption, and at 50 °C, 65 °C, and 80 °C for desorption for 90 min each (For E/A = 0.3, 110 min at 50 °C).

Figure 6 shows the results of CO_2_ adsorption and desorption tests. At E/A = 0.3, despite the thick fiber diameter of 865 nm, the adsorption rate is very fast due to the high adsorption performance of primary amines. Similarly, E/A = 0.4 has a thick fiber diameter of 661 nm, but the adsorption rate is fast. However, at E/A = 0.3 to 0.4, where a large amount of primary amine remains, the amount of desorption at 50 °C (from 990 min to 1100 min) is low due to the high absorbability of the primary amine, reducing the low-temperature desorption property and showing that adsorption and desorption performance are trade-offs. On the other hand, for the E/A = 0.5 sample, which contains only secondary amines, the desorption characteristic at 50 °C is high, with an analyzed saturated desorption ratio of 52%. In addition, for the sample with a high E/A of 0.55, some tertiary amines existing in the sample are not involved in adsorption from secondary amines, so the amount of adsorption is reduced. Since the remaining adsorption ratio after desorption at 50 °C is equivalent to E/A = 0.5, it is considered that the tertiary amines do not have any positive influence on desorption characteristics.

For the retention ratio of the adsorbed amount after the heat treatment for 200 h at 85 °C, higher E/A had a higher retention ratio. Reduction of the retention ratio is thought to be due to the decrease in the adsorption site by the reduction of primary amines, which are vulnerable to oxidation. Oxidation resistance can be improved by the formation of secondary amines through a chemical reaction with epoxy.

Furthermore, the low-temperature desorption at 50 °C exhibited a high desorption ratio of approximately 50%, with E/A ranging from 0.5 to 0.55, clearly indicating that secondary amines demonstrate superior low-temperature desorption compared to primary amines. Additionally, at 65 °C, the desorption properties were nearly equivalent within the E/A range of 0.4 to 0.55, thereby confirming the advantage of secondary amination at low temperatures of 50 °C. These findings suggest that secondary amination of amines not only enhances oxidation resistance but also contributes significantly to low-temperature desorption properties. To achieve optimal adsorption and desorption performance, it is important to note that the glass transition temperature (Tg) of the sorbent needs to be close to the operating temperature during DAC. Generally, when the sorbent temperature exceeds Tg, molecular mobility increases, facilitating CO_2_ desorption. Therefore, a lower Tg is desirable for CO_2_ adsorption; however, if Tg is too low, the handling of the solid sorbent becomes difficult. Consequently, E/A = 0.5 is considered to offer the best balance between adsorption and desorption performance, with a Tg being the closest to the adsorption temperature and high oxidative stability. In conclusion, the control of E/A to convert all primary amines to secondary amines was verified to be essential.

### 3.3. The Effect of the Degree of Saponification of PVA

Since amine and epoxy alone do not possess sufficient spinnability, poly(vinyl alcohol) (PVA) was selected as a spinnability-enhancing material. PVA exhibits varying properties depending on its molecular weight and degree of saponification. The degree of saponification represents the proportion of saponification during PVA synthesis, with higher values indicating a greater conversion of acetic acid groups to hydroxyl groups. It is well established that a higher degree of saponification in PVA correlates with increased crystallinity and higher solution viscosity. Accordingly, the materials listed in Table 5 were electrospun with an applied voltage of 10 kV and 8 cm between the syringe and the collector, under inline heating conditions at a temperature of 120 °C. Using the obtained webs, the effect of the degree of saponification of PVA on the adsorption characteristics and heat resistance durability of AE/PVA nanofibers was investigated.

**Figure 7:**
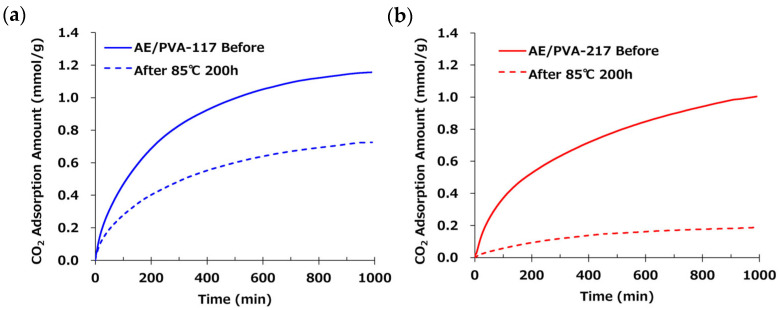
(**a**) CO_2_ adsorption profiles of AE/PVA–117 before and after heat treatment. The solid blue line represents the original AE/PVA–117 sample, while the dashed blue line corresponds to the sample after heat treatment at 85 °C for 200 h. (**b**) CO_2_ adsorption profiles of AE/PVA–217 before and after heat treatment. The solid red line represents the original AE/PVA–217 sample, while the dashed red line corresponds to the sample after heat treatment at 85 °C for 200 h.

**Figure 8:**
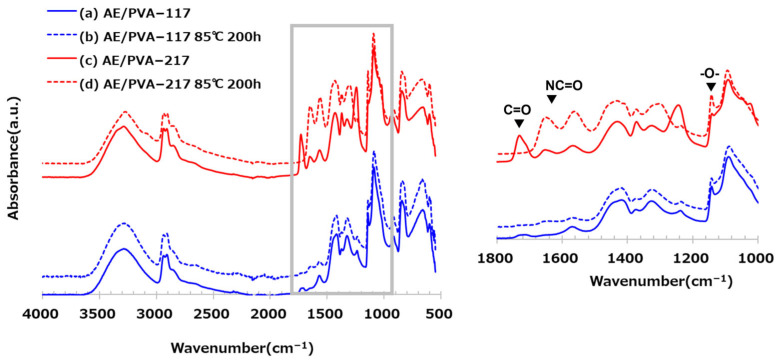
FT-IR spectra of AE/PVA–117 (blue) and AE/PVA–217 (red) before and after heat treatment. Solid lines represent the original samples, and dashed lines correspond to samples after heat treatment at 85 °C for 200 h. Enlarged FT-IR spectra of AE/PVA–117 and AE/PVA–217 in the range of 1800–1000 cm^−1^ are inserted to highlight characteristic absorption bands. Peaks corresponding to C=O (~1700 cm^−1^), NC=O (~1500 cm^−1^), and –O– (~1200 cm^−1^) are annotated.

**Figure 9:**
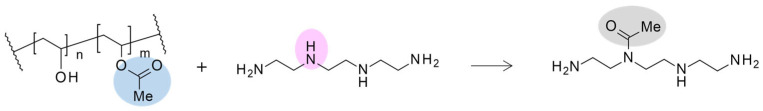
The proposed reaction mechanism between the acetate groups in the PVA-based polymer and the amine groups in the curing agent. The reaction leads to the formation of amide bonds, R–C(=O) –N(–R’)–R”, as suggested by FT-IR spectral changes observed after heat treatment.

Table 6 shows nanofibers with PVA saponification degrees of 99% (PVA-117) and 88% (PVA-217), as well as the results of their adsorption tests when AE/PVA nanofibers were prepared. Even when using PVA with different degrees of saponification, AE/PVA nanofibers with almost the same diameter of 350 nm could be prepared. As for adsorption performance, the adsorption amount after 990 min is 1.16 mmol/g for PVA-117 and 1.00 mmol/g for PVA-217, and the adsorption performance can be said to be almost the same. Regarding the thermal durability, as shown in Figure 7, the adsorption test for the webs before and after being stored at 85 °C for 200 h revealed that the adsorption characteristics differed greatly, depending on the degree of saponification of PVA. Compared to AE/PVA-117, AE/PVA-217 shows a significant decrease in the amount of saturation adsorption after the heat treatment. As shown in Figure 8, FT-IR measurements of the fiber web before and after heat resistance test exhibited that AE/PVA-217 showed a large decrease in the peak of the acetyl group at 1750 cm^−1^, and a large increase in the peak of the amide group around 1600 cm^−1^. It was speculated that the acetyl group of PVA reacted with the secondary amine and was amidated, as shown in Figure 9. As a result, it is considered that the secondary amine, which is the adsorption site, decreased, and the amount adsorbed decreased. Based on these results, it was decided to use PVA-117, which has a low acetic acid group or a high degree of saponification as a spinning material. The selected PVA-117, which does not react with primary or secondary amines, does not inhibit CO_2_ adsorption, is water-soluble and inexpensive, and is ideal for AE/PVA nanofibers as a material to impart spinnability.

## 4. Discussion

This study revealed that CO_2_ adsorption behavior in AE/PVA nanofibers is governed by multiple inter-related factors—namely, fiber diameter, the epoxy-to-amine ratio (E/A), and the degree of PVA saponification. These findings elucidate the design principles for optimizing sorbent performance in direct air capture (DAC) applications.

The influence of fiber diameter was particularly evident in the CO_2_ adsorption rate. Finer nanofibers exhibited accelerated adsorption due to their higher specific surface area and enhanced exposure of amine functionalities. The correlation between fiber diameter and adsorption kinetics, including adsorption half-time, underscores the advantage of producing homogeneously thin fibers. However, the measured BET surface areas were significantly lower than theoretical predictions, suggesting that morphological factors such as fiber adhesion and diameter distribution limit accessible surface area, an issue warranting further process refinement.

The modulation of the E/A demonstrated a classic trade-off in amine-based sorbents. Lower E/A, which retains more primary amines, yielded fast adsorption but poor low-temperature desorption characteristics, while higher E/A (up to 0.5) facilitated secondary amine formation, achieving better desorption behavior and oxidative stability. E/A = 0.5 was identified as the optimal condition, balancing kinetic performance, desorption efficiency, and material stability.

Additionally, the degree of PVA saponification played a critical role in long-term thermal durability. The presence of residual acetate groups in lower-saponified PVA led to amide bond formation with secondary amines during heat treatment, as evidenced by FT-IR analysis. This undesirable side reaction decreased the available amine sites for CO_2_ capture. Consequently, highly saponified PVA-117 was deemed the superior spinnability enhancer due to its chemical inertness and compatibility. When the degree of PVA saponification is low, residual acetate groups may react with amines to form amide bonds. This side reaction is expected to occur more extensively at lower E/A ratios where a higher number of free amine groups are present, potentially leading to greater thermal degradation. In contrast, at higher E/A ratios, the number of reactive primary and secondary amines is reduced due to crosslinking, which may suppress such degradation

Collectively, the interplay between formulation and processing conditions has a decisive impact on sorbent performance. Tailoring these parameters allows for strategic enhancement of DAC efficiency, underscoring the material’s potential for scalable deployment.

## 5. Conclusions

In this study, we systematically explored the effects of fiber diameter, epoxy-to-amine ratio (E/A), and the degree of PVA saponification on the CO_2_ adsorption behavior of electrospun AE/PVA nanofibers. Our findings highlight three key factors that govern the performance of these solid amine-based adsorbents. The results confirmed the following: (1) Fiber Morphology: Thinner fibers significantly enhanced CO_2_ adsorption kinetics by increasing the accessible surface area, facilitating faster gas diffusion and interaction with active sites. (2) Chemical Composition: An optimized E/A ratio of 0.5 provided a balanced trade-off between adsorption capacity, desorption efficiency, and thermal durability, ensuring effective and repeatable CO_2_ capture cycles. (3) Material Stability: Highly saponified PVA effectively suppressed side reactions with amines, thereby improving the long-term chemical and thermal stability of the nanofibers.

Moreover, the AE/PVA nanofiber sheets demonstrated favorable thermal stability and resistance to volatilization and leaching, minimizing the risk of equipment corrosion. Their self-supporting sheet form enables straightforward integration into filter-type modules, which is advantageous for practical deployment in direct air capture (DAC) systems. While fiber–fiber adhesion may reduce the BET surface area and slightly slow initial adsorption rates, it contributes positively to mechanical robustness by preventing fiber unraveling. This trade-off supports the development of scalable, durable, and high-performance sorbents.

Overall, this work provides a foundational framework for the rational design of next-generation solid amine adsorbents, emphasizing the importance of simultaneous control over fiber morphology, chemical composition, and thermal compatibility for effective DAC applications.

## Figures and Tables

**Table 1:** The degree of saponification, degree of polymerization, and molecular weight of poly(vinyl alcohol) (PVA) samples used in this study.

	Degree of Saponification (%)	Degree of Polymerization	Molecular Weight
PVA-117	99	1700	76,000
PVA-217	88	1700	83,000

**Table 2:** SEM images of electrospun fibers prepared under different electrospinning conditions by varying the applied voltage and the distance between the syringe and the collector. Diameter of the spun fibers, the CO_2_ adsorption amount obtained through the adsorption test, and the SEM images with ×1000, ×5000, and ×10,000 magnifications for electrospun AE/PVA fiber are also shown.

	No.1	No.2	No.3
Voltage (V)	15	10	15
Distance (cm)	12	8	8
Voltage/Distance (V/cm)	1.25	1.25	1.875
Fiber Diameter (nm)	383	420	454
CO_2_ Adsorption Amount (mmol/g)	1.67	1.56	1.49
Adsorption Half Time (min)	28.3	45.7	110
SEM ×1 k	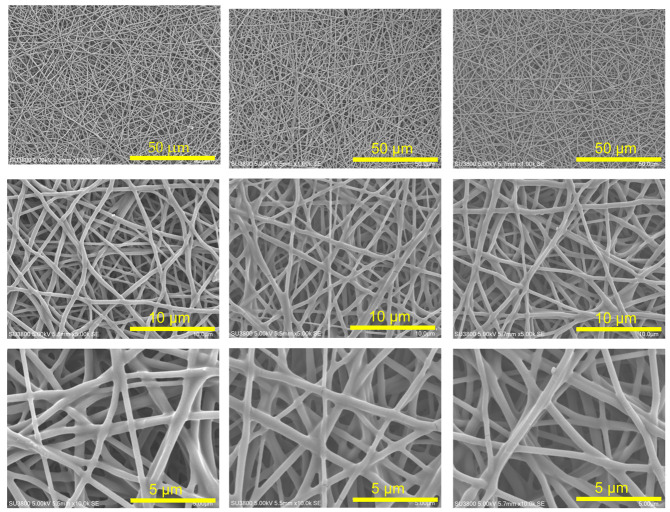		
SEM ×5 k			
SEM ×10 k			

**Table 3:** Compositions of epoxy resin systems with varying epoxy-to-amine ratios (E/A). The table includes the amounts of EDE and T-X, SP-006, and the concentrations of PVA in aqueous solution. The weight ratio of AE to the total of AE and PVA is also listed.

E/A	0.3	0.4	0.5	0.55
EDE (g)	0.8	0.8	0.8	0.8
T-X (g)	0.2	0.2	0.2	0.2
SP-006 (g)	2.67	2.00	1.60	1.45
PVA 7 wt% Aq. Solution (g)	33.0	27.0	23.4	22.1
EDE: T-X	8:2	8:2	8:2	8:2
AE: PVA Aq. Solution	1:9	1:9	1:9	1:9
AE/(AE + PVA) (wt%)	61	61	61	61

**Table 4:** Fiber properties of epoxy-based materials prepared with varying epoxy-to-amine ratios (E/A). The table summarizes fiber diameter, glass transition temperature (Tg), CO_2_ adsorption amount after 990 min at 20 °C, predicted saturation values of adsorption based on the Avrami model, and desorption ratios after 90 min each at 50, 65, and 80 °C. (For E/A = 0.3, 110 min at 50 °C). Heat resistance (retention ratio of adsorption amount after treatment at 85 °C for 200 h) is also included. SEM images at magnifications of ×3000 and ×5000 show the fiber morphologies under each condition.

E/A	0.3	0.4	0.5	0.55
Fiber Diameter (nm)	865	661	481	424
Tg (°C)	5.1	10.3	19.0	24.8
CO_2_ Adsorption Amount (mmol/g)	1.86	1.7	1.6	1.42
Prediction Saturation Value of Adsorption (mmol/g)	1.91	1.84	1.64	1.59
Desorption Ratio at 50 °C (%)	37	44	52	48
Desorption Ratio at 65 °C (%)	90	92	94	93
Desorption Ratio at 80 °C (%)	100	100	100	100
Heat Resistance (%)	54	67	74	—
SEM ×3 k	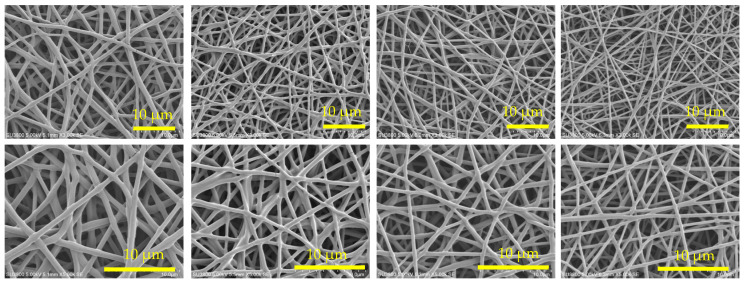			
SEM ×5 k				

**Table 5:** Compositional parameters of AE/PVA-117 and AE/PVA-217 systems. The table summarizes the amounts of ODE, T-C, TETA, and PVA solution, the ratio of ODE to T-C, the epoxy-to-amine ratio (E/A), the type of PVA used, the ratio of AE to PVA aqueous solution, and the weight percentage of AE in the resultant AE + PVA mixture.

	AE/PVA-117	AE/PVA-217
ODE (g)	0.8	0.8
T-C (g)	0.2	0.2
TETA (g)	1.6	1.6
8 wt% PVA Aq. Solution (g)	23.4	23.4
ODE: T-C	8:2	8:2
E/A	0.5	0.5
PVA (saponification deg.)	PVA-117 (99%)	PVA-217 (88%)
AE: PVA 8 wt% Aq. Solution	1:9	1:9
AE/(AE + PVA)	58%	58%

**Table 6:** A comparison of fiber properties between AE/PVA-117 and AE/PVA-217 systems. The table summarizes fiber diameter, CO_2_ adsorption amount, predicted saturation value, and heat resistance (retention ratio of adsorption amount after treatment at 85 °C for 200 h). SEM images at magnifications of ×3000 and ×10,000 are shown for each sample.

	AE/PVA-117	AE/PVA-217
Fiber Diameter (nm)	362	326
CO_2_ Adsorption Amount (mmol/g)	1.16	1.00
Prediction Saturation Value (mmol/g)	1.25	1.34
Heat Resistance (%)	68%	15%
SEM ×3 k	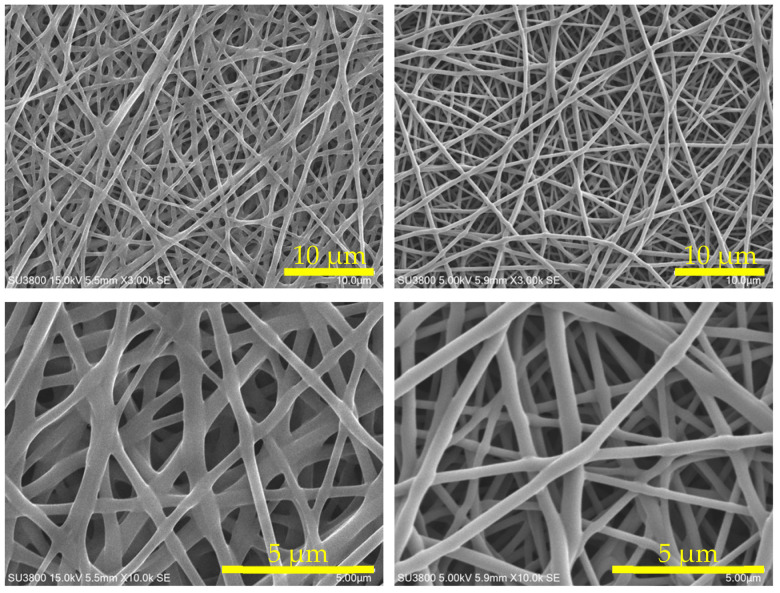	
SEM ×10 k		

## Data Availability

The data supporting the findings of this study are available from the corresponding author upon reasonable request.
